# Numerical investigation of mixed physical barriers for saltwater removal in coastal heterogeneous aquifers

**DOI:** 10.1007/s11356-023-31454-z

**Published:** 2023-12-18

**Authors:** Sobhy R. Emara, Asaad M. Armanuos, Bakenaz A. Zeidan, Tamer A. Gado

**Affiliations:** Irrigation and Hydraulics Engineering Department, Faculty of Engineering, Tanta University, Tanta, Egypt

**Keywords:** Cutoff wall, Subsurface dam, SEAWAT, Saltwater interface, And Stratified aquifer

## Abstract

Saltwater intrusion is a prevalent global environmental issue that detrimentally impacts coastal groundwater aquifers. This problem is exacerbated by climate change and increased groundwater abstraction. Employing physical barriers proves effective in mitigating saline water intrusion. In this study, a validated numerical simulation model is utilized to assess the impact of aquifer stratification on the effectiveness of mixed physical barriers (MPBs) and their response to structural variations. Additionally, the performance of MPBs was compared with that of single physical barriers in a laboratory-scale aquifer. Three different configurations were replicated, comprising two stratified aquifers (HLH and LHL) and a homogenous reference aquifer (H). The results demonstrate that MPBs are efficient in decreasing the saltwater penetration length in the investigated cases. The reductions in penetration length were up to 65% in all cases. The removal efficacy of residual saline water for MPBs exceeded that of the subsurface dam by 2.1–3.3 times for H, 2.1–3.6 times for HLH, and 8.3 times for LHL conditions, while outperforming the cutoff wall by 38–100% for H, 39–44% for HLH, and 2.7–75% for LHL. These findings are of importance for decision-makers in choosing the most appropriate technique for mitigating saline water intrusion in heterogeneous coastal aquifers.

## Introduction

Seawater intrusion (SWI), or contamination of coastal groundwater with saline seawater, poses a main challenge for coastal regions worldwide, particularly when groundwater (GW) sources serves as the primary source of freshwater (Shalby et al. 2023a, 2023b). The severity of SWI's impact is primarily driven by the excessive extraction of groundwater (Werner et al. 2013). Climate change plays a central role in exacerbating saltwater intrusion, manifesting in both direct and indirect effects. The direct effect is evident in rising sea levels, while the indirect effect involves reduced rainfall in certain areas, leading to decreased natural groundwater recharge. These combined effects result in an inland extension of the saltwater wedge, which is further intensified by the overexploitation of groundwater (Ketabchi et al. 2016; Abdoulhalik and Ahmed 2017a; Emara et al. 2023a, 2023b).

The existence of heterogeneity within aquifers can exert varying degrees of influence on solute transport mechanisms and flow dynamics, contingent on the degree of aquifer heterogeneity (Werner et al. 2013). Dagan and Zeitoun (1998) studied the influence of aquifer heterogeneities on the location of the saltwater-freshwater interface using the sharp interface technique. Lu et al. (2013) conducted laboratory investigations on steady-state saltwater intrusion in heterogeneous aquifers with various stratification patterns. They observed that the scenario with a homogeneous aquifer exhibited a significantly greater extent of the saltwater wedge compared to the layered scenarios. Further insights into the influence of aquifer stratification on SWI were provided by Etsias et al. (2021), who examined three homogeneous strata and six stratified aquifers through numerical simulations and laboratory experiments. Their findings revealed that when the lower permeability layer is positioned either above or below the more permeable layer, the mixing zone expands, whereas in aquifers with higher permeability strata, the mixing zone becomes thinner. In a more recent investigation, Gao et al. (2022) employed variable-density coupling with reactive transport numerical models to explore and quantify the dynamic changes in salinity and nitrate levels within layered heterogeneous coastal aquifers in the presence of cut-off walls. The comprehensive simulations conducted in their study suggest that nitrate accumulation is primarily governed by aquifer heterogeneities.

Seawater-contaminated groundwater becomes unsuitable for residential, agricultural, or industrial use (Zhang et al. 2019; Yu et al. 2019). To safeguard groundwater quality in coastal zones by preventing SWI, researchers have developed a range of practical engineering solutions. These include optimizing the layout of pumping wells (Yang et al. 2018; Liu et al. 2019; Fan et al. 2020; Ranjbar et al. 2020), utilizing positive hydraulic barriers like artificial recharge by infiltration or injection (Clark et al. 2014; Hussain et al. 2016; Motallebian et al. 2019; Armanuos et al. 2020a), employing negative hydraulic barriers, such as pumping saltwater inland (Javadi et al. 2012, 2015; Mehdizadeh et al. 2019), and constructing underground physical barriers (Kaleris and Ziogas 2013; Abd-Elaty et al. 2019; Armanuos et al. 2019, 2020b).

Relocating pumping wells inland to establish a proper seaward hydraulic gradient is a common practice to combat seawater intrusion. However, meticulous planning of well locations is crucial. This approach can be constrained by factors such as land availability and potential conflicts with other projects. The use of high-quality water, like desalinated water, for recharge barriers, can be costly and may face challenges due to water scarcity in certain regions. Extraction barriers protect aquifers by creating a seaward hydraulic gradient but may result in excessive freshwater extraction, diminishing aquifer storage. Controlled pumping rates can make this method suitable for temporary management in cases of limited intrusion or as a preliminary step in salinity control before applying other methods (Hussain et al. 2019).

Hydraulic and physical barriers are two major approaches for preventing SWI in aquifers (Werner et al. 2013; Zhang et al. 2019). When compared to other methods, underground physical barriers (PB) efficiently manage SWI while also preserving freshwater supplies (Yan et al. 2021). Despite their relatively high cost, many underground barriers have been proposed to control SWI in various locations across the world, such as Muğla, Turkey (Onder and Yilmaz 2005), Florida, United States of America (Abd-Elaty et al. 2019), and the Zakaki area, Cyprus (Armanuos et al. 2020b).

**Fig. 1:**
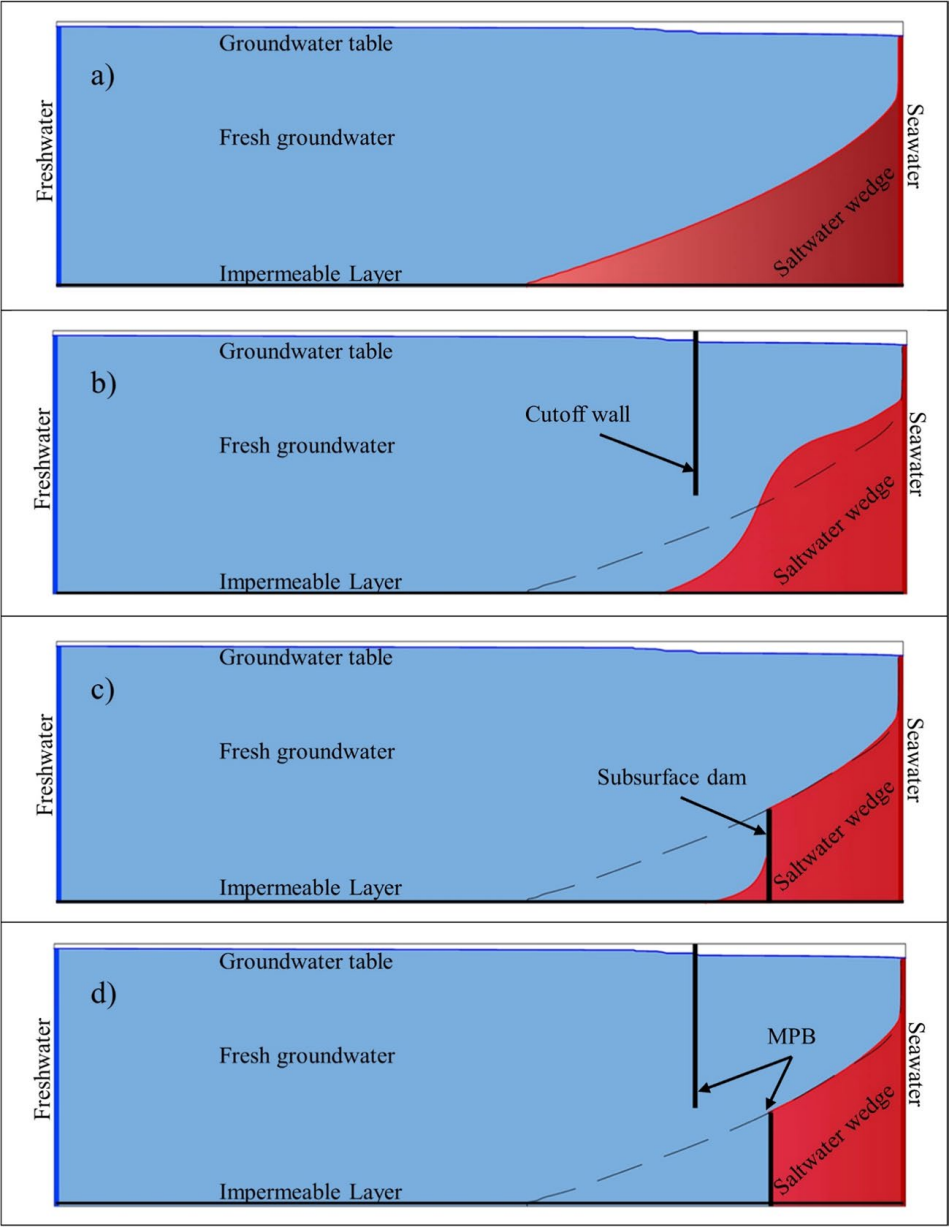
Diagrams depicting the Saltwater Intrusion (**a**), Cutoff Wall (**b**), Subsurface Dam (**c**), and Mixed Physical Barrier (MPB) (**d**). The dashed line denotes the initial position of the saltwater wedge

Subsurface dams and cutoff walls represent two prevalent physical barriers in addressing saltwater intrusion. A subsurface dam is typically situated in the lower part of an aquifer, leaving a space at its upper portion to allow the natural flow of freshwater towards the sea (Fig. 1b). This arrangement effectively hinders the encroachment of saltwater towards the land (Luyun et al. 2009; Hussain et al. 2019). On the other hand, cutoff walls, which extend from the aquifer's surface to a certain depth, demonstrate enhanced effectiveness, particularly when deployed in proximity to the shoreline and with considerable penetration depth (Luyun et al. 2011; Armanuos 2017; Armanuos et al. 2020b).

Several numerical simulations and laboratory experiments have been conducted to assess the effectiveness of subsurface barriers in controlling SWI (Senthilkumar and Elango 2011; Abdoulhalik and Ahmed 2017a; Armanuos 2017; Robinson et al. 2018; Armanuos et al. 2019). Kaleris and Ziogas (2013) conducted numerical investigations that examined various parameters such as wall shape, hydraulic characteristics, and aquifer properties, evaluating their impact on reducing saltwater intrusion and safeguarding groundwater withdrawal. Using numerical simulations and laboratory experiments, Abdoulhalik and Ahmed (2017b) demonstrated that the effectiveness of subsurface dams (Fig. 1c) in preventing saltwater intrusion and removing residual saline water is strongly connected to the stratification degree of the aquifers. Zheng et al. (2022) investigated the dynamic behavior and desalination process of intruding seawater following the construction of a cutoff wall. This research utilized a numerical model validated through laboratory experiments. The quantity of remaining saltwater primarily depends on both the depth and hydraulic conductivity of the cutoff wall, while the timeframe for seawater retreat is notably influenced by the wall's placement.

The highly concentrated saltwater that remains in the aquifers upstream of the physical barrier once the barrier is in place is referred to as residual saltwater. This issue poses a significant challenge, as it restricts the use of fresh groundwater in coastal areas (Luyun et al. 2009). As a result, a crucial aspect of SWI control involves addressing this lingering saline water within the aquifer. However, research suggests that the saltwater wedge trapped by the physical barrier remains stationary because the residual saltwater cannot be easily flushed out (Oswald and Kinzelbach 2004). Using numerical and experimental models, Luyun et al. (2009) discovered that the residual seawater retained by the underground barrier can be gradually removed by groundwater flow. Nevertheless, this removal process takes substantially longer than the duration of saltwater intrusion. Zheng et al. (2021) were the first to reveal the mechanism for eliminating residual saltwater in the presence of subsurface dams. They also examined how the structure of the dam and the characteristics of the aquifer influence the effectiveness of removing the remaining saltwater retained upstream of the dam. Their findings revealed that under conditions of high-concentration gradients, the lower-concentration mixing zone serves as a crucial pathway for saltwater to migrate over the subsurface dam and reach the saltwater edge. In essence, residual saltwater is continuously disseminated from the higher-concentration mixing zone to the lower-concentration mixing zone.

In addition to the previously mentioned physical barriers, Abdoulhalik et al. (2017) introduced a novel approach known as a mixed physical barrier (MPB), which involves combining a semi-permeable dam wall with a cutoff wall (Fig. 1d). This innovative combination proved to be highly effective in significantly reducing the extent of saltwater intrusion by facilitating the upward movement of saltwater toward the coastline. To investigate the applicability of MPB, Gao et al. (2021) studied the dynamics of groundwater flow and residual saltwater subsequent to the construction of an MPB under homogeneous conditions. Their research provided compelling evidence of the advantages associated with employing MPBs, particularly in terms of groundwater discharge and removing residual saltwater.

The inevitability and widespread occurrence of coastal aquifer heterogeneity have been well-established. Notably, the presence of stratified heterogeneity, as documented in various instances (Gao et al. 2022), exerts a substantial influence on both groundwater flow and the intrusion of saltwater. Previous research has predominantly utilized physical barriers to control saltwater intrusion in homogeneous media (Gao et al. 2021; Zheng et al. 2022). In this research, a pioneering effort was made to investigate the combined impacts of stratified heterogeneity and the implementation of mixed physical barriers on groundwater flow and the enrichment of saltwater intrusion within coastal unconfined aquifers. To achieve this, a series of 2D numerical simulations were executed, incorporating various configurations of mixed physical barriers, considering the effects of aquifer stratification. The outcomes of this research are anticipated to offer valuable guidance for designing mixed physical barriers tailored to the specific challenge of saltwater intrusion management in stratified coastal aquifers.

## Materials and methods

In this study, the SEAWAT model was used to simulate groundwater variable-density flow based on the numerical finite difference GW flow simulation model MODFLOW. To the best of our knowledge, the current research is the first attempt to explore the combined effects of a cutoff wall and a subsurface dam on the removal of residual saline water in a heterogeneous condition. Previous investigations, such as the study by Gao et al. (2021), only examined the impact of MPBs in homogeneous conditions. The efficacy of mixed physical barriers in controlling SWI in heterogeneous soil formations was a central focus of this study. The dimensions of the experimental unconfined aquifer used by Gao et al. (2021) served as the basis for validating the numerical model. Various scenarios were generated within a vertical 2D cross-section domain to assess the efficiency of residual saline water removal following the installation of MPBs. To quantify this efficiency, parameters such as the overall efficacy (η), the reduction rate of the saltwater wedge length (R_L_) upstream of the MPBs, and the average correlation coefficient (R^2^) were employed to compare and evaluate the agreement between numerical and experimental results. The study also introduced dimensionless parameters, including the dimensionless subsurface dam height (H_d_*), dimensionless cutoff wall depth (H_c_*), and dimensionless MPB spacing (L_s_*), to determine the most effective dimensions for MPBs.

### Conceptual Model

SEAWAT was used in the current study to develop numerical models for simulating solute transport in GW flow. The primary objective was to investigate how the presence of mixed physical barriers (MPBs) impacts the removal of residual saline water in heterogeneous soil formations. The study examined the mechanisms of saltwater intrusion and retreat both before and after the installation of MPBs. To streamline the problem and facilitate the modeling process, several assumptions were made:

1. The concentration of salt mass is the sole factor influencing variations in fluid density.
2. The aquifer media is consistently saturated.
3. The groundwater is incompressible.

**Fig. 2:**
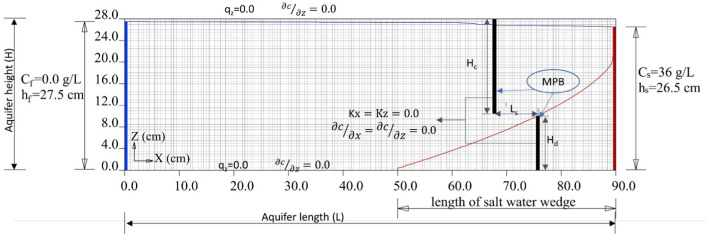
Boundary conditions for the numerical simulations

The simulation area in the SEAWAT model is a vertical 2D section with dimensions of 90.0 cm by 28.0 cm, and it is uniformly discretized into a finite-difference mesh using quadratic elements with grid spacing of Δx × Δz = 0.5 cm × 0.5 cm (Fig. 2). To ensure numerical stability, the grid spacing and dispersivity must satisfy the Péclet number criterion (Voss and Souza 1987). The Péclet number (${{\text{Pe}}}_{{\text{m}}}\approx \Delta {\text{x}}/{\mathrm{\alpha }}_{{\text{l}}}$) is a dimensionless parameter that quantifies the proportion of local advective transport to local diffusion and dispersive transport, where ${\mathrm{\alpha }}_{{\text{l}}}$ is the longitudinal dispersivity of the porous media. For this study, a longitudinal dispersivity (${\mathrm{\alpha }}_{{\text{l}}}$) of 0.15 cm was assigned to the porous media, a value commonly used in laboratory experiments (Sun et al. 2019; Chang et al. 2019; Gao et al. 2021). In this study, both the dispersivity value and the grid spacing satisfy the Péclet number criterion (${{\text{Pe}}}_{{\text{m}}}=\frac{0.5\mathrm{ cm}}{0.15\mathrm{ cm}}=3.33<4$.0), indicating that the numerical setup maintains sufficient stability.

In the numerical simulations, the boundary conditions were set as follows:

- On the right-hand side, there was a saltwater boundary with a fixed salt concentration (C_s_) of 36 g/L. This boundary had a constant head (${{\text{h}}}_{{\text{s}}}$) of 26.50 cm.
- On the left side, a freshwater boundary was established with a fixed salt concentration (C_f_) of 0 g/L.
- The densities of saltwater and freshwater were 1025 g/L and 1000 g/L, respectively.
- A hydraulic gradient of 0.0111 was applied to the system by adjusting the freshwater level (${{\text{h}}}_{{\text{f}}}$=27.5 cm), resulting in a head difference of 1.0 cm. This value falls within the typical range of hydraulic gradients employed in prior laboratory-scale research of a similar nature (Chang and Clement 2012; Abdoulhalik and Ahmed 2017a).
- No-flow boundary conditions were imposed at both the top and bottom boundaries of the aquifer.

Figure 2 represents the boundary conditions of the numerical simulations, where the MPB dam and wall, depicted as black bars, were located 14.0 and 22.0 cm away from the saline water boundary, respectively. The parameters for the wall depth, dam height, and spacing are denoted as H_c_, H_d_, and L_s_, respectively.

**Fig. 3:**
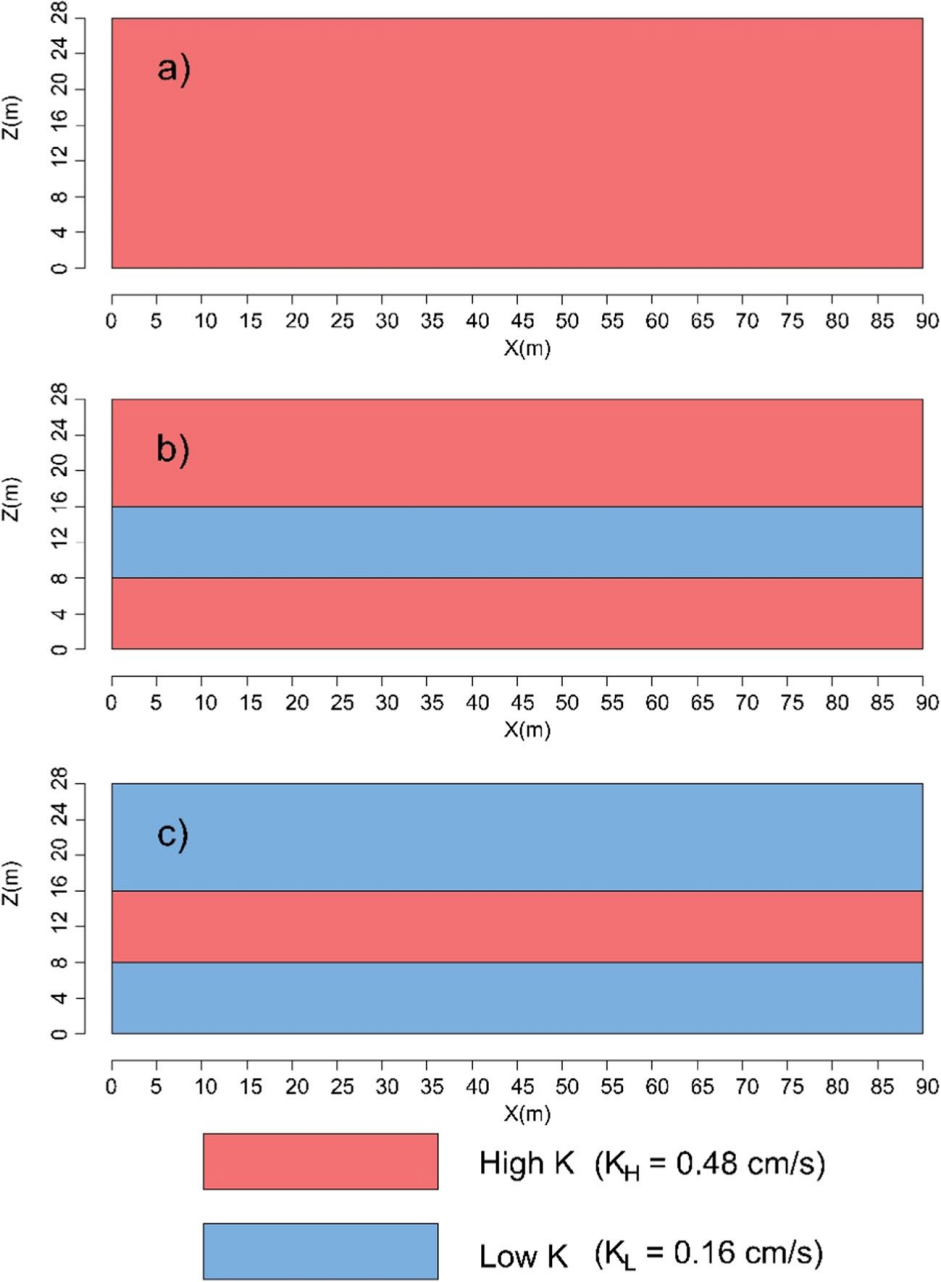
Schematic design of the investigated aquifer settings: **a** homogeneous, **b** stratified HLH, and **c** stratified LHL

Two different stress periods were established for each aquifer simulation to represent both the SWI process and the subsequent removal process following MPB construction. The duration of these stress periods was determined through preliminary runs to ensure that the saltwater interface reached a stable state. The first stress period had a length of 7.0 h, while the second lasted for 10 h. To investigate the influence of MPBs on SWI, three different aquifers were simulated, including a homogenous aquifer and two stratified aquifers. The stratified aquifers consisted of three layers with varying hydraulic conductivity (K) arranged as high K – low K – high K and low K – high K – low K), as shown in Fig. 3. The first heterogeneous scenario involved the creation of a low hydraulic conductivity layer (K = 0.16 cm/s) in the central portion of the aquifer (Abdoulhalik et al. 2020; Ahmed et al. 2022). In contrast, the second heterogeneous scenario was characterized by low hydraulic conductivity layers established in both the upper and lower sections of the aquifer, enabling the assessment of heterogeneous effects in distinct flow conditions. As a benchmark, simulations were conducted for the three aquifers without MPBs to establish an initial stable saltwater interface. The simulation procedures for both the baseline and MPB scenarios were nearly identical. To simulate MPB in the model, the cells encompassed by the dam and wall were set as inactive (Luyun et al. 2009; Gao et al. 2021).

The three layers of the stratified aquifers have specific thicknesses: the middle and lower layers are 8 cm thick, while the upper layer, which contains the water table, has a thickness of 12 cm. In the reference MPB construction, the dam height was set at 10 cm, measured from the aquifer bottom, the wall depth was established at 24 cm, measured from the ground surface, and the barrier spacing was maintained at 8 cm. In the simulation, only one variable was modified at a time for a comparison with the reference MPB scenario. A total of 60 sets of simulations were conducted, including three different aquifers, with 20 simulations for each aquifer. These simulations were carried out based on the model parameters outlined in Table 1. Table 2 presents a summary of the specific cases investigated for each aquifer, outlining the variations in model parameters for the different scenarios.

**Table 1:** Model parameters utilized in the numerical simulations (modified after Gao et al. (2021))

Parameter	Value	Unit
Aquifer length (L)	90	cm
Aquifer height (H)	28	cm
Freshwater head (h_f_)	27.5	cm
Saltwater head (h_s_)	26.5	cm
Freshwater density (ρ_f_)	1000	g/L
Saltwater density (ρ_s_)	1025	g/L
Salt concentration in freshwater (C_f_)	0.0	g/L
Salt concentration in saltwater (C_s_)	36.0	g/L
Investigated aquifer settings	case H; case HLH, case LHL	-
Hydraulic conductivity (K_H_) High K layer	0.48	cm/s
Hydraulic conductivity (K_L_) Low K layer	0.16	cm/s
Porosity (n)	0.4	-
Longitudinal dispersivity (α_L_)	0.15	cm
Transversal dispersivity (α_T_)	0.015	cm
Specific yield (S_y_)	0.2	-
Depth of cutoff wall (H_c_)	12, 16, 20, 24, 26	cm
Height of subsurface dam (H_d_)	7, 10, 13, 16, 19	cm
Spacing of MPB (L_s_)	2, 4, 6, 8,10	cm
Hydraulic gradient (J)	1.11	%
Dimensionless subsurface dam height (H_d_*$=\frac{{{\text{H}}}_{{\text{d}}}}{{{\text{h}}}_{{\text{s}}}}$)	0.264, 0.377, 0.491, 0.604, 0.717	-
Dimensionless cutoff wall depth (H_c_*$=1-\frac{{\text{H}}-{{\text{H}}}_{{\text{c}}}}{{{\text{h}}}_{{\text{s}}}}$)	0.396, 0.547, 0.698, 0.849, 0.925	-
Dimensionless MPB spacing (L_s_*$=\frac{{{\text{L}}}_{{\text{s}}}}{{\stackrel{`}{{\text{L}}}}_{{\text{i}}}}$)	0.051, 0.103, 0.154, 0.205, 0.257	-

${\stackrel{`}{{\text{L}}}}_{{\text{i}}}$ is the residual saltwater wedge initial length measured from the subsurface dam

**Table 2:** Summary of the investigated cases for each aquifer

No	Cutoff wall depth (H_c_, cm)	Subsurface dam height (H_d_, cm)	MPB Spacing (L_s_, cm)
1	-	-	-
2	26	10	8
3	24	10	8
4	20	10	8
5	16	10	8
6	12	10	8
7	24	7	8
8	24	13	8
9	24	16	8
10	24	19	8
11	24	10	10
12	24	10	6
13	24	10	4
14	24	10	2
15	24	-	-
16	20	-	-
17	16	-	-
18	-	7	-
19	-	10	-
20	-	13	-

### Evaluation methods

To evaluate the effectiveness of residual saline water removal subsequent to the installation of MPBs in the inland aquifer upstream of the MPBs, key performance indicators, specifically the overall efficacy (η) and the saltwater wedge repulsion ratio (${{\text{R}}}_{{\text{L}}}$), were employed (Luyun et al. 2011; Armanuos et al. 2019; Motallebian et al. 2019; Gao et al. 2021). The indices were calculated as follows:

1 $$\upeta =\frac{{{\text{L}}}_{{\text{i}}}-{\text{L}}}{{{\text{L}}}_{{\text{i}}}}$$

2 $${{\text{R}}}_{{\text{L}}}=\frac{{\stackrel{`}{{\text{L}}}}_{{\text{i}}}-\stackrel{`}{{\text{L}}}}{{\stackrel{`}{{\text{L}}}}_{{\text{i}}}}$$

where ${\stackrel{`}{{\text{L}}}}_{{\text{i}}}$ and ${{\text{L}}}_{{\text{i}}}$ represent the initial length of the residual saltwater wedge measured from the subsurface dam and the saltwater boundary on the right side, respectively, and ${\text{L}}$ and $\stackrel{`}{{\text{L}}}$ refer to the length of the residual saline water wedge after MPB construction measured from the subsurface dam and the saltwater boundary on the right side, respectively. To track the position of the toe, measurements were taken every ten minutes until the saltwater wedge reached a steady-state condition, defined as the point when the variation in saltwater penetration length remained less than 1.0 mm for a continuous 30-min period. The location of the toe was determined using the 50% saltwater salinity isoline, specifically at 18,000 mg/L, which is also used to delineate the geometry of the saltwater-freshwater interface (Ahmed et al. 2022).

## Results and discussions

To investigate the efficacy of mixed physical barriers in mitigating SWI in heterogeneous soil formations, several scenarios were studied using the SEAWAT model, as described below. First, the SEAWAT model was validated by comparing the numerical results with the experimental data from Gao et al. (2021). Then, the influences of cutoff wall depth, subsurface dam height, and MPB spacing were investigated in three different aquifers, including a homogenous aquifer and two different layered aquifers. Finally, the performance of MPB was compared with that of a single physical barrier.

### Model validation

**Fig. 4:**
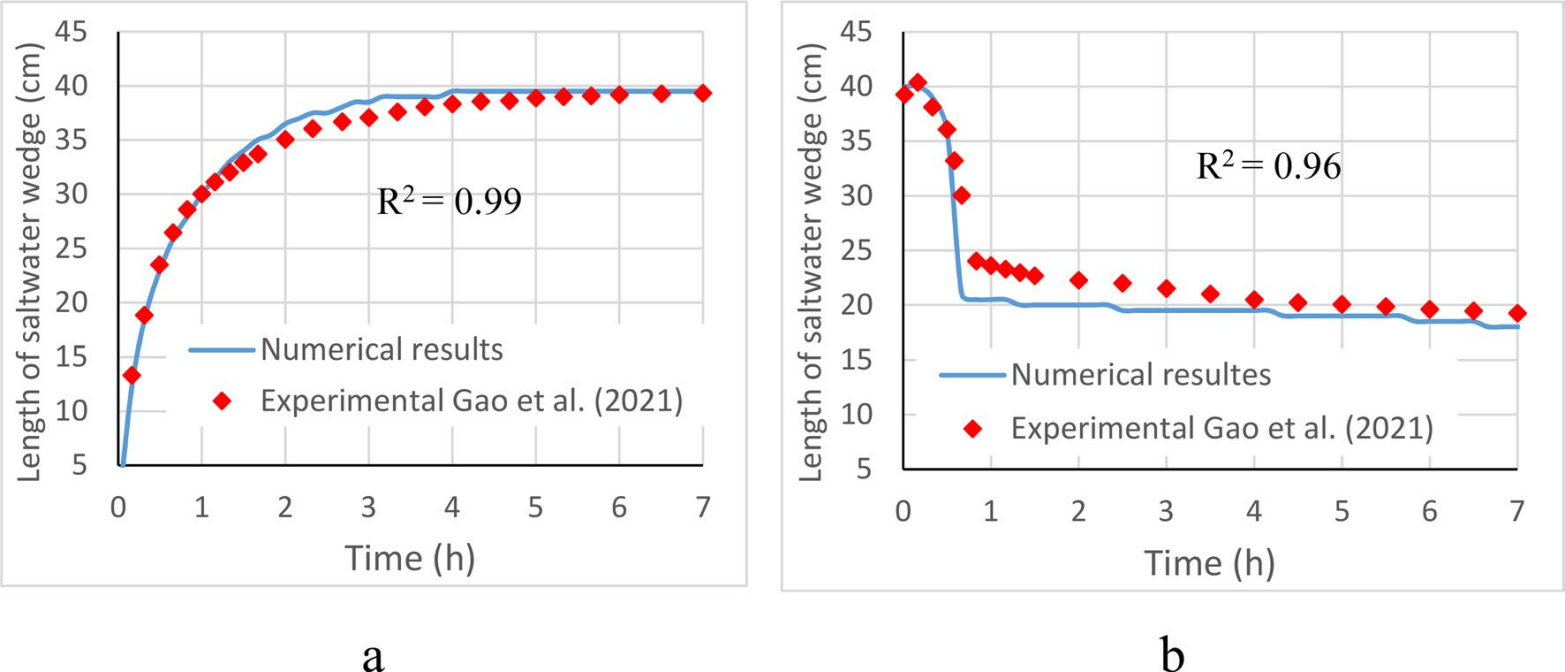
Comparison of the numerical and experimental (Gao et al. 2021) temporal change of saltwater wedge length before **a** and after **b** MPB fitting for the reference structure (H_d_ = 10 cm, H_c_ = 24 cm, and L_s_ = 8 cm) in the homogeneous case

The temporal change of saltwater wedge length, as observed in the experiment carried out by Gao et al. (2021), was used to validate the numerical model, particularly in the context of a homogeneous configuration, as shown in Fig. 4. The figure depicts the consistency between the numerical and experimental results before (a) and after (b) the installation of MPBs for the reference structure (H_d_ = 10 cm, H_c_ = 24 cm, and L_s_ = 8 cm). In Fig. 4, the length of the 50% saltwater salinity isoline (equivalent to 18000 mg/L) is shown, a widely utilized criterion in numerical simulations to identify the extent of the saltwater wedge (e.g., Strack et al. 2016; Abd-Elaty et al. 2019; Gao et al. 2021). The average significant correlation coefficient (R^2^) was calculated to evaluate the match between the numerical and experimental results. The numerical simulations demonstrate a strong agreement with the experimental data, yielding R^2^ values of 0.997 and 0.967 for the pre- and post-MPB installation cases, respectively.

### Mechanism of residual saltwater removal

**Fig. 5:**
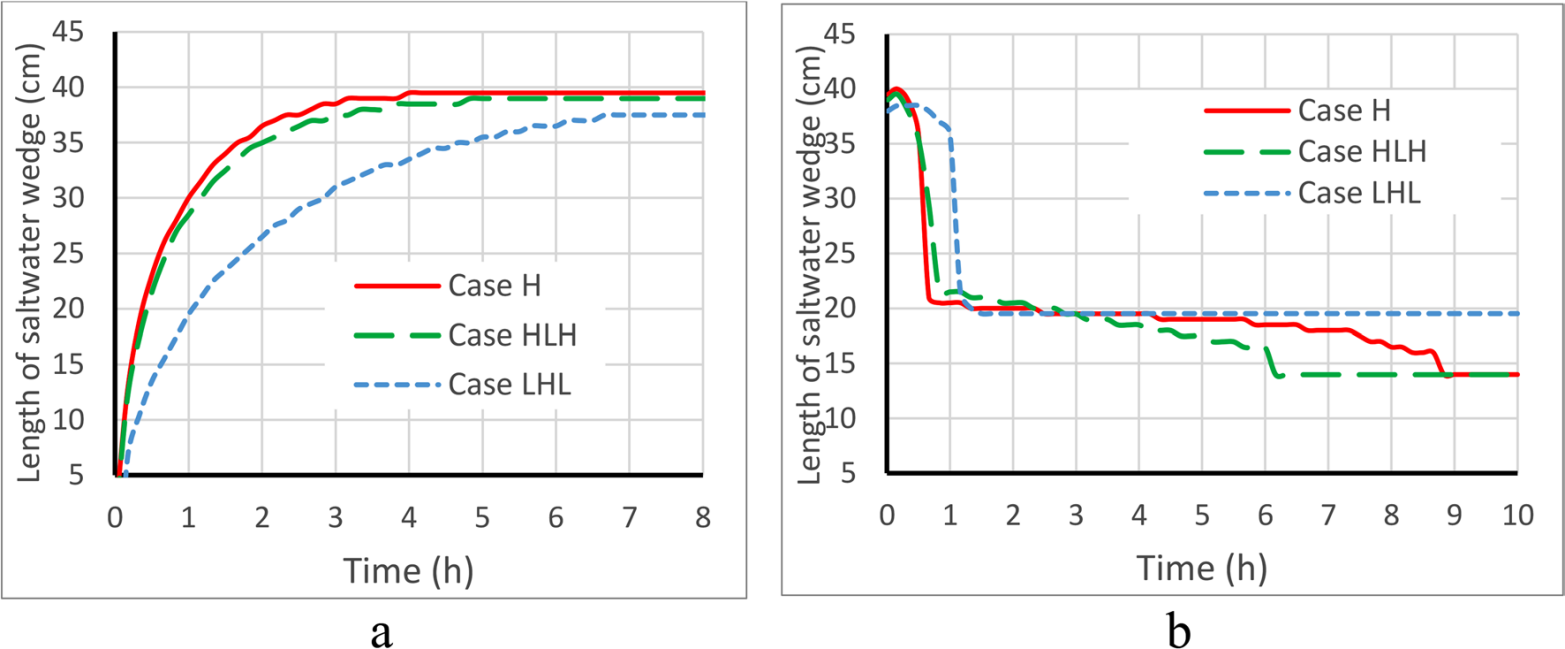
The temporal change of saltwater wedge length before **a** and after **b** MPB fitting for the reference structure (H_d_ = 10 cm, H_c_ = 24 cm, and L_s_ = 8 cm)

Following the successful validation of the numerical model, the subsequent results were obtained through simulations. The time-dependent evolution of saltwater wedge length for the three cases (H, HLH, and LHL) is illustrated in Fig. 5, for both pre- and post-MPB installation. In Fig. 5a, it is evident that the saltwater wedge length experiences an initial rapid expansion over time before stabilizing. The advancement of saltwater in the homogeneous and HLH aquifers occurs at a faster rate than in the LHL case. Once the seawater intrusion reaches a steady state, the removal process starts with the construction of the MPBs. According to the removal rate of the residual saltwater, as shown in Fig. 5b, the removal process can be divided into two distinct stages. During the first stage, within the first hour of the removal process, the MPBs exhibit high efficiency, resulting in a rapid reduction of the saltwater wedge penetration length by 48.1% (H), 44.9% (HLH), and 43.4% (LHL). This demonstrates the significant efficacy of removing residual saltwater during this first stage. Additionally, the area of the mixing zone (AMZ) between the 10% and 90% salinity contour lines of seawater (as defined by Gao et al. 2021) is significantly increased in the first stage (Fig. 7). Upon the implementation of the MPBs, the freshwater flow becomes constrained to the region beneath the cutoff wall. Initially, this region is predominantly occupied by saltwater. Consequently, there is an instantaneous expansion of the mixing zone, spanning between the 10% and 90% isolines of seawater salinity. This expansion signifies an enhanced level of interaction and blending between saltwater and freshwater, a phenomenon consistent with the findings of Kaleris and Ziogas (2013). This widening of the mixing zone, coupled with the influence of the fresh groundwater flow, results in a rapid retreat of the saltwater wedge and an efficient removal of residual saltwater during the first stage. These observations align closely with the research conducted by Gao et al. (2021) in the context of a homogeneous aquifer scenario. In the second stage, after 1.0 h, the rate of residual saltwater removal is significantly slower in all cases until reaching a steady state. Finally, the residual saltwater penetration length recedes, leading to a decline in removal efficiency and mixing intensity (Fig. 5). For case HLH, the residual saltwater wedge held in the inland aquifer upstream of the subsurface dam is removed after 6.0 h, while in the homogeneous case, it takes 9.0 h. In the case of LHL, the residual saltwater wedge shortens by 48.7% in about 1.5 h, after which it reaches a steady state at that position.

**Fig. 6:**
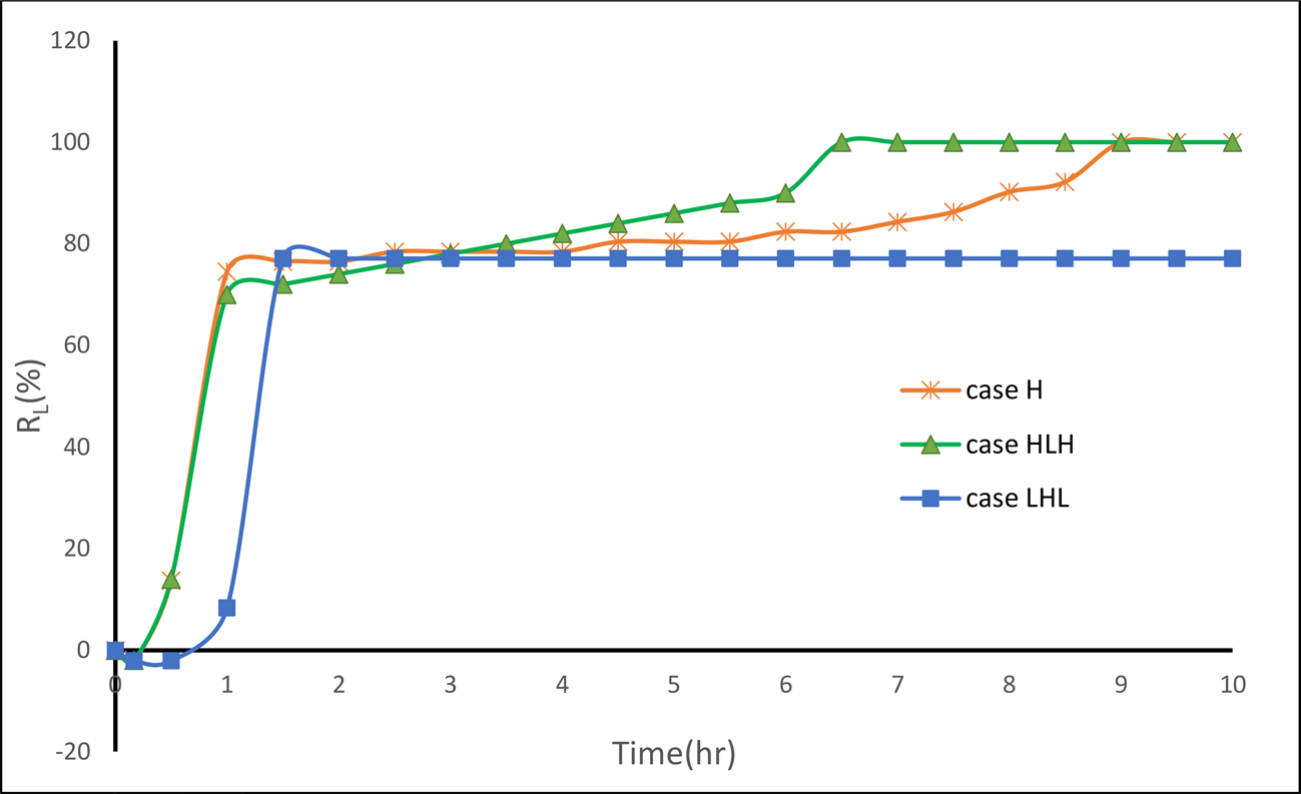
Transient repulsion ratio R_L_ value for MPB with the reference structure (H_d_ = 10 cm, H_c_ = 24 cm, and L_s_ = 8 cm)

**Fig. 7:**
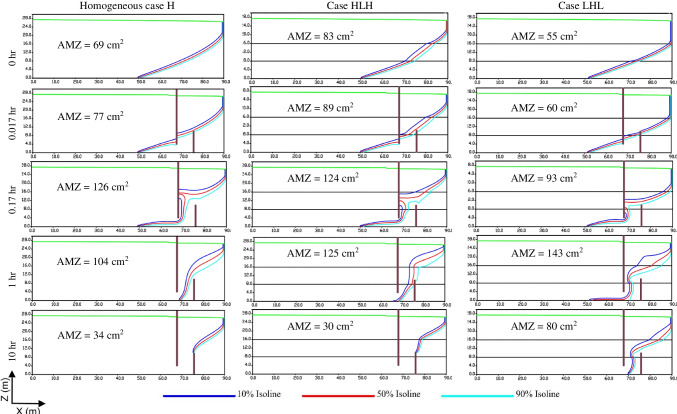
Dynamic changes of concentration field and the area of the mixing zone (AMZ) after MPB installation with reference dimensions for various periods for the three distinct aquifer settings

The repulsion ratio $({{\text{R}}}_{{\text{L}}})$ exhibited a decreasing trend during the first 10 min of the removal process (Fig. 6). The negative value of ${{\text{R}}}_{{\text{L}}}$ implies that the penetration length of the residual saltwater wedge expanded rather than decreased. This phenomenon can be attributed to the constrained space available for the MPBs, which in turn impeded the rate of fresh groundwater discharge. As a result, it took a longer period to reestablish the equilibrium between saltwater and freshwater, as explained by Gao et al. (2021) (Fig. 7).

### Impacts of subsurface dam height

In this part of the study, the depth of the cutoff wall ${({\text{H}}}_{{\text{c}}})$ and the spacing of MPB (L_s_) remained fixed, set equal to the reference case, while the height of the subsurface dam (H_d_) was subjected to simulations with different values (7, 10, 13, 16, and 19 cm). The dynamic behavior of residual saltwater after MPB construction is shown in Fig. 15, Fig. 16, and Fig. 17, for the cases H, HLH, and LHL respectively, with subsurface dam heights of 7, 10, and 13 cm for different periods. When the subsurface dam was taller in all aquifer conditions studied, it posed a greater challenge for highly concentrated saline water to refill the residual saline water (Fig. 15c, Fig. 16c, and Fig. 17c). The dam height remained above the 90% isoline, and as a result, the residual saltwater with a salinity exceeding 10.0% was completely eliminated after 10 h. At this point, the seawater wedge had been pushed seaward to the other side of the subsurface dam.

**Fig. 8:**
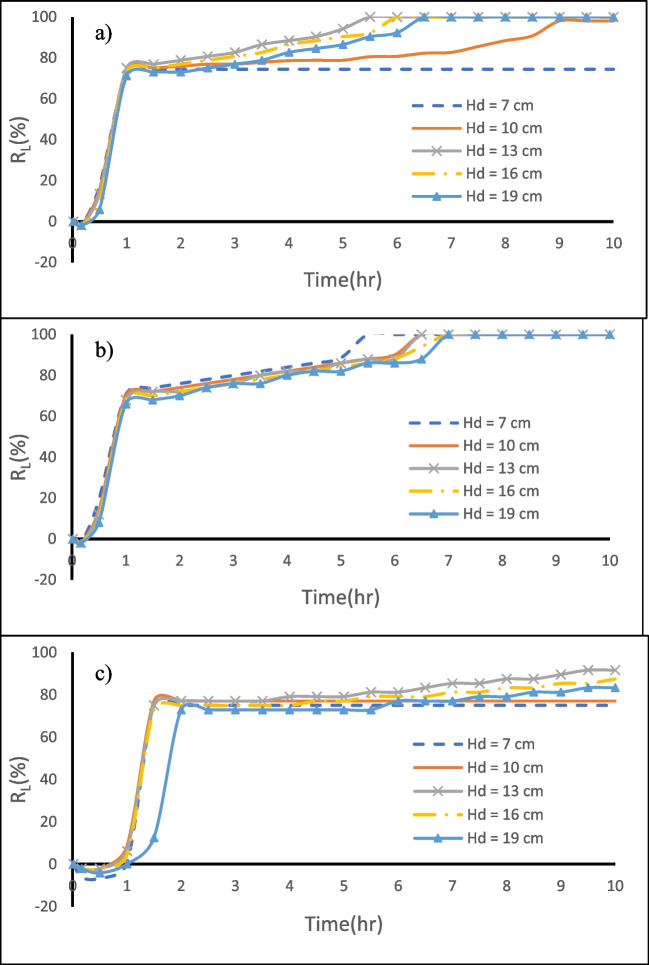
Impact of the subsurface dam height on MPBs repulsion ratio for **a** case H, **b** case HLH, and **c** case LHL

Figure 8 shows the ${{\text{R}}}_{{\text{L}}}$ change over time for various dam heights in the three different aquifer configurations under investigation. The ${{\text{R}}}_{{\text{L}}}$ values for ${{\text{H}}}_{{\text{d}}}$ = 7.0, 10.0, 13.0, 16.0, and 19.0 cm after 10 h from MPB construction for the investigated cases are provided in Table 3. In the first two cases (H and HLH), approximately one hour following the installation of the MPB, the repulsion ratio ${({\text{R}}}_{{\text{L}}})$ climbed to over 75% and then continued to rise at a diminishing rate until it approached stabilization, reaching ${{\text{R}}}_{{\text{L}}}=100\mathrm{\%}$ after removing all the residual saltwater from the aquifer upstream of the subsurface dam. For case H (homogeneous aquifer), the dam height of 13 cm was the first to attain a steady state after 5.5 h from the start of the removal process. In contrast, the increase in dam height had no significant effect on the efficiency of the MPB in the case HLH.

**Table 3:** The ${R}_{L}$ and $\eta$ values for ${H}_{d}$= 7.0, 10.0, 13.0, 16.0, and 19.0 cm after 10 h of MPB construction for case H, case HLH, and case LHL

Case	H_d_ = 7 cm		H_d_ = 10 cm		H_d_ = 13 cm		H_d_ = 16 cm		H_d_ = 19 cm	
	R_L_ ( %)	η ( %)	R_L_ ( %)	η ( %)	R_L_ ( %)	η ( %)	R_L_ ( %)	η ( %)	R_L_ ( %)	η ( %)
H	74.4	48.4	100.0	64.6	100.0	64.6	100.0	64.6	100.0	64.4
HLH	100.0	64.1	100.0	64.1	100.0	64.1	100.0	64.1	100.0	64.1
LHL	75.0	47.4	77.1	48.7	91.7	57.9	87.5	55.3	83.3	52.6

In the third case (LHL), the ${{\text{R}}}_{{\text{L}}}$ value increased to more than 75% after approximately 1.1 h from the construction of MPBs. However, it's worth noting that the efficacy of the MPBs in removing residual saltwater decreased as the dam height increased. This decline in efficiency was attributed to the significant increase in the height of the subsurface dam, which extended the path for the drainage of residual saltwater (Table 3). Notably, the existence of the bottom low conductivity layer (LHL) was found to impede the fresh GW discharge in the bottom section of the aquifer. This restriction had the effect of reducing the repulsion capacity and prolonging the time required for removal.

**Fig. 9:**
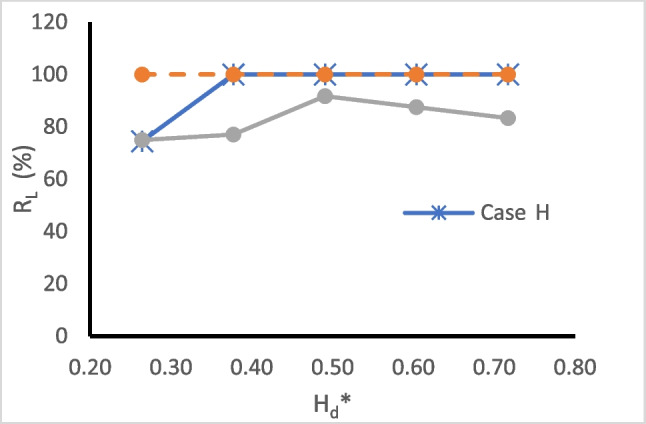
The relation between the repulsion ratio ($${R}_{L})$$ and dimensionless subsurface dam height (H_d_*) for case H, case HLH, and case LHL

Figure 9 presents the relationship between the repulsion ratio $({{\text{R}}}_{{\text{L}}})$ and dimensionless subsurface dam height (H_d_*) for the three investigated aquifers. H_d_* is obtained by dividing the height of the subsurface dam (H_d_) by the saltwater head (h_s_). The curves in the graph illustrate that the MPBs with an H_d_* value greater than or equal to 0.377 can completely remove the residual saline water in case H. For case HLH, all H_d_* values examined resulted in a repulsion ratio of 100%. In the case of LHL, ${{\text{R}}}_{{\text{L}}}$ values exhibit a positive correlation with H_d_* values spanning from 0.26 to 0.5 and a negative correlation with H_d_* values ranging from 0.5 to 0.72. It appears that an MPB with H_d_* equal to 0.5 achieves the maximum repulsion ratio for all investigated cases.

### Impacts of cutoff wall depth

To investigate the impact of the cutoff wall depth on removal efficacy, the height of the subsurface dam (H_d_) and the MPB spacing (L_s_) values were maintained at reference values, while several cutoff wall depths (12, 16, 20, 24, and 26 cm) were applied, as illustrated in Table 1. The temporal progression of residual saltwater removal following the construction of MPB with various cutoff wall depths, spanning 0.017, 0.17, 1, and 10 h, is shown in Fig. 18, Fig. 19, and Fig. 20 for the cases H, HLH, and LHL respectively.

**Fig. 10:**
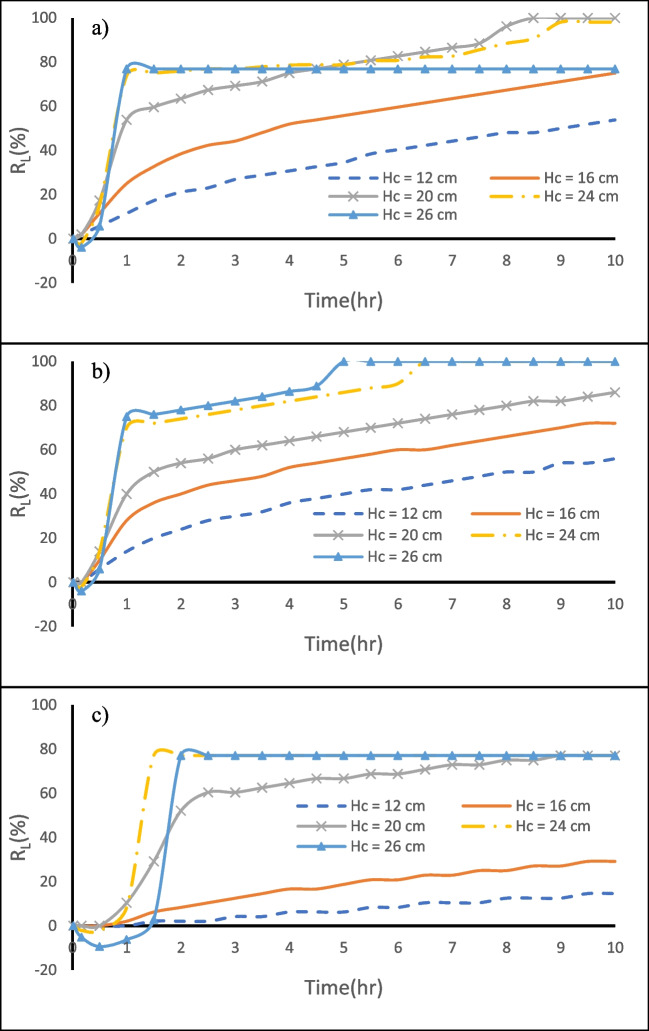
Influences of cutoff wall depth on MPB performance, **a** case H, **b** case HLH, and **c** case LHL

Notably, in case H (Fig. 18**)**, it is evident that following 1 h of MPB construction, the mixing zone extended from 69 to 130 cm^2^, 104 cm^2^, and 86 cm^2^ for cutoff wall depths equal 26, 24, and 20 cm, respectively. In the case of HLH (Fig. 19), after 1 h of MPB construction, the mixing zone extended from 83 to 165 cm2, 125 cm2, and 102 cm2 for cutoff wall depths equal 26, 24, and 20 cm, respectively. Likewise, in the context of LHL (Fig. 20), following 1 h of MPB construction, the mixing zone expanded from 55 to 170 cm2, 143 cm2, and 97 cm2 for cutoff wall depths equal 26, 24, and 20 cm, respectively. These results underscore the impact of the cutoff wall, which enhances the speed of groundwater flow beneath the cutoff wall and augments the intensity of mixing between freshwater and saltwater. These effects become more pronounced as the cutoff wall depth increases. The evolution in R_L_ values over time is depicted in Fig. 10. For all the investigated aquifers, when the depth of the cutoff wall was relatively large, the repulsion ratio value exhibited a clear and significant increase, as observed, for example in the first hour in the case of ${{\text{H}}}_{{\text{c}}}$= 24 cm. However, the growth rate of R_L_ rapidly dropped with the reduction of the cutoff wall's depth, indicating that the MPB's capacity to remove residual saltwater was less effective. The ${{\text{R}}}_{{\text{L}}}$ values associated with cutoff wall depths ${{\text{H}}}_{{\text{c}}}$ = 26, 24, 20, 16, and 12 cm after 10 h of MPB construction for the investigated cases are provided in Table 4.

**Table 4:** The ${R}_{L}$ and $\eta$ values for ${H}_{c}$= 26, 24, 20, 16, and 12 cm after 10 h of MPB installation for case H, case HLH, and case LHL

Case	H_c_ = 26 cm		H_c_ = 24 cm		H_c_ = 20 cm		H_c_ = 16 cm		H_c_ = 12 cm	
	R_L_ ( %)	η ( %)	R_L_ ( %)	η ( %)	R_L_ ( %)	η ( %)	R_L_ ( %)	η ( %)	R_L_ ( %)	η ( %)
H	76.9	50.0	100.0	64.6	100.0	64.6	74.5	48.1	52.9	34.2
HLH	100.0	64.1	100.0	64.1	86.0	55.1	72.0	46.2	56.0	35.9
LHL	77.1	48.7	77.1	48.7	77.1	50.0	29.2	18.4	14.6	9.2

**Fig. 11:**
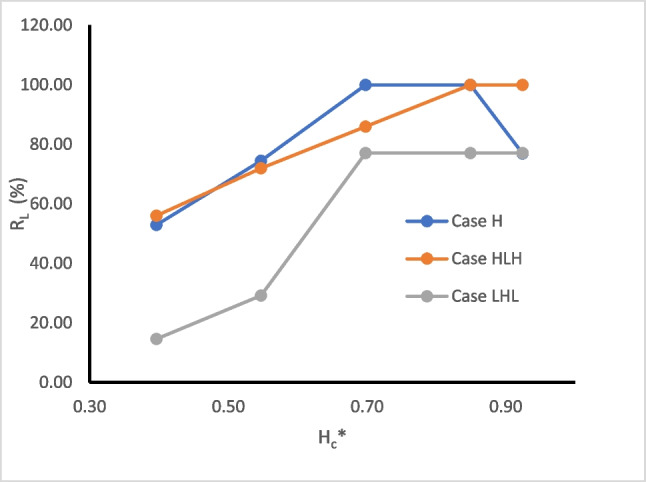
The relation between the repulsion ratio ${(R}_{L})$ and dimensionless cutoff wall depth (H_c_*) for case H, case HLH, and case LHL

Figure 11 presents the relationship between the repulsion ratio ${({\text{R}}}_{{\text{L}}})$ and the dimensionless cutoff wall depth (H_c_^*^) for the three investigated aquifers. H_c_^*^ is calculated using the formula ${{\text{H}}}_{{\text{c}}}^{*}=1-\frac{{\text{H}}-{{\text{H}}}_{{\text{c}}}}{{{\text{h}}}_{{\text{s}}}}$, where H represents the aquifer thickness, H_c_ is the cutoff wall depth, and h_s_ the saltwater head. Increasing the dimensionless cutoff wall depth results in an increase in the repulsion ratio of saltwater intrusion. This relationship holds for a range of H_c_^*^ values ranging from 0.4 to 0.85, with the achieved repulsion ratio $({{\text{R}}}_{{\text{L}}})$ ranging from 14.6% to 100%. The curves illustrate that the MPB with H_c_^*^ value equal to 0.85 appears to attain the maximum repulsion ratio in all the cases under investigation.

### Impacts of MPB spacing

In this section of the study, the height of the subsurface dam ${({\text{H}}}_{{\text{d}}})$ and the depth of the cutoff wall $({{\text{H}}}_{{\text{c}}})$ remained consistent with the reference case, while various spacing values for MPB $({{\text{L}}}_{{\text{s}}})$ (2.0, 4.0, 6.0, 8.0, and 10.0 cm) were utilized to investigate the impact of MPB spacing on saltwater removal efficiency. The position of the MPB was determined based on the location of the subsurface dam, which was held constant, while the placement of the wall was adjusted to alter the distance between the barriers. The dynamic behavior of residual saltwater following the construction of MPBs with 10, 8, and 6 cm spacing over intervals of 0.017, 0.17, 1, and 10 h is shown in Fig. 21, Fig. 22, and Fig. 23 for case H, case HLH, and case LHL, respectively.

**Fig. 12:**
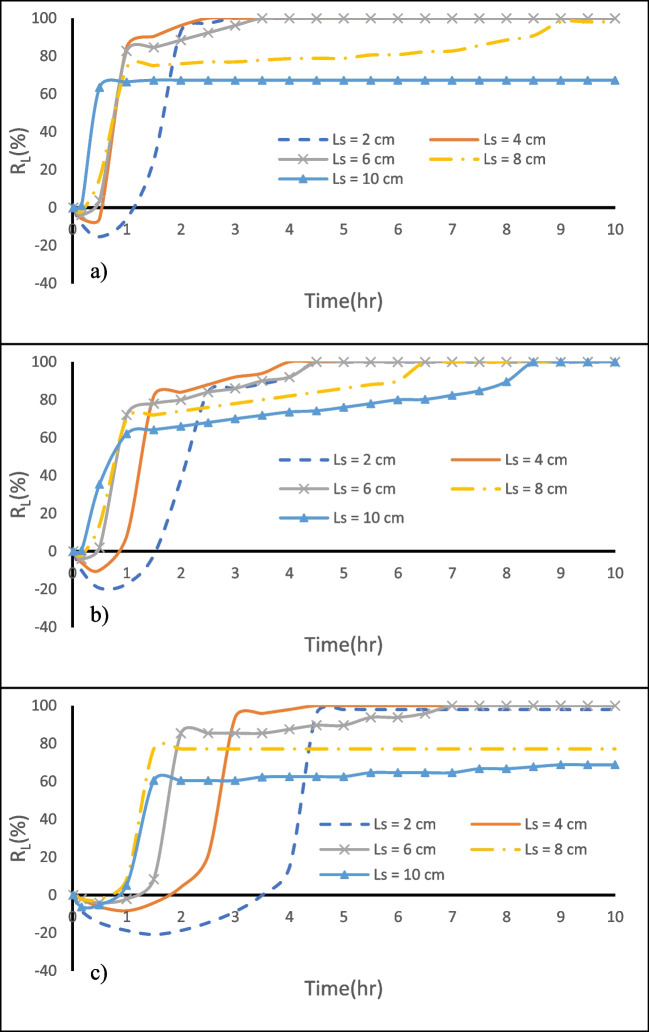
Influences of MPB spacing on MPB performance. **a** case H, **b** case HLH, and **c** case LHL

In case H, the wedge of the residual saltwater shrunk from 25.5 to 6.5 cm within the first hour for ${{\text{L}}}_{{\text{s}}}$= 8 cm. When the MPB spacing was reduced to 6 cm or when the cutoff wall was shifted 2 cm closer to the coast (Fig. 21c), the residual saline water wedge shrank from 25.5 to 4.5 cm within the first hour. This is a result of freshwater flowing beneath the cutoff wall, which effectively washed over the residual saltwater wedge more comprehensively. In the case of HLH, the wedge of the residual saltwater shortened from 25 to 7.5 cm within the first hour when the MPB spacing was 8 cm. Likewise, when the spacing was further reduced to 6 cm, the wedge shrunk to 7 cm within the first hour. Notably, the residual saltwater held upstream of the subsurface dam was eliminated after 4.5 h for ${{\text{L}}}_{{\text{s}}}$ = 6 cm and after 6.5 h with an 8 cm spacing (Fig. 12b). For the LHL case, the residual saltwater wedge diminished from 24 to 5.5 cm in about 1.5 h when the MPB spacing was 8 cm, eventually reaching a steady state at this position. On the other hand, when the MPB spacing was reduced to 6 cm, the residual saltwater trapped upstream of the subsurface dam was washed away after 7.0 h (Fig. 12c).

The rate of ${{\text{R}}}_{{\text{L}}}$ growth exhibited a slowdown after 1.0 to 1.1 h for L_s_ = 8 cm. This pattern was consistent across all cases, indicating that for L_s_ = 8 cm, the rates of ${{\text{R}}}_{{\text{L}}}$ increase began to decelerate after 1.0 to 1.1 h. In contrast, with smaller spacing, the rates of ${{\text{R}}}_{{\text{L}}}$ increase remained relatively high until the wedge of residual saltwater vanished. As a result, the residual saltwater retained upstream of the subsurface dam was effectively eliminated within 10 h, underscoring the exceptional removal efficiency of this particular MPB setup (Table 5).

**Table 5:** The ${R}_{L}$ and $\eta$ values for ${L}_{S}$= 10, 8, 6, 4, and 2 cm after 10 h of MPB installation for case H, case HLH, and case LHL

Case	L_s_ = 10 cm		L_s_ = 8 cm		L_s_ = 6 cm		L_s_ = 4 cm		L_s_ = 2 cm	
	R_L_ ( %)	η ( %)	R_L_ ( %)	η ( %)	R_L_ ( %)	η ( %)	R_L_ ( %)	η ( %)	R_L_ ( %)	η ( %)
H	76.3	43.1	100.0	64.6	100.0	64.6	100.0	64.6	100.0	64.6
HLH	100.0	64.1	100.0	64.1	100.0	64.1	100.0	64.1	100.0	64.1
LHL	68.8	43.4	77.1	48.7	100.0	63.2	100.0	63.2	97.9	61.8

**Fig. 13:**
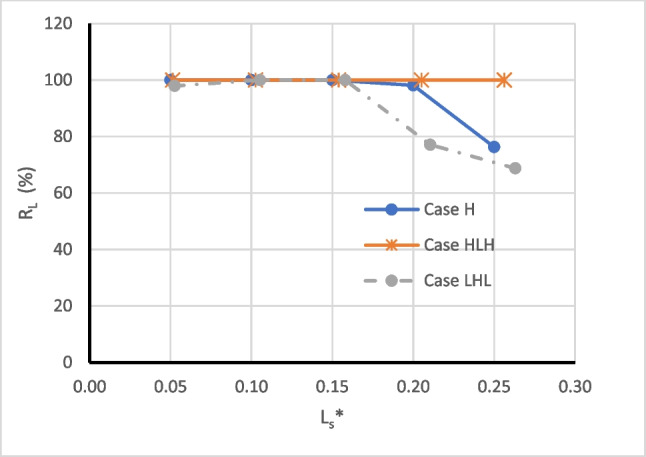
The relation between the repulsion ratio ${(R}_{L})$ and dimensionless MPB spacing (L_s_*) for case H, case HLH, and case LHL

Figure 13 presents the relationship between the repulsion ratio $({{\text{R}}}_{{\text{L}}})$ and dimensionless MPB spacing (L_s_^*^) for the three investigated aquifers. L_s_^*^ is calculated by dividing the MPB spacing (L_s_) by the initial length of the residual saltwater wedge measured from the subsurface dam (${\stackrel{`}{{\text{L}}}}_{{\text{i}}}$). The curves in the graph illustrate that the MPB with an L_s_^*^ value less than or equal to 0.15 seems to achieve the maximum repulsion ratio in all investigated cases.

### MPB versus a single barrier

To evaluate the effectiveness of MPBs, their performance was compared with that of single barriers, consisting of a cutoff wall and a subsurface dam with varying lengths, over a 10-h stress period. R_L_ was utilized to assess the capability of the physical barriers in removing residual saline water.

**Fig. 14:**
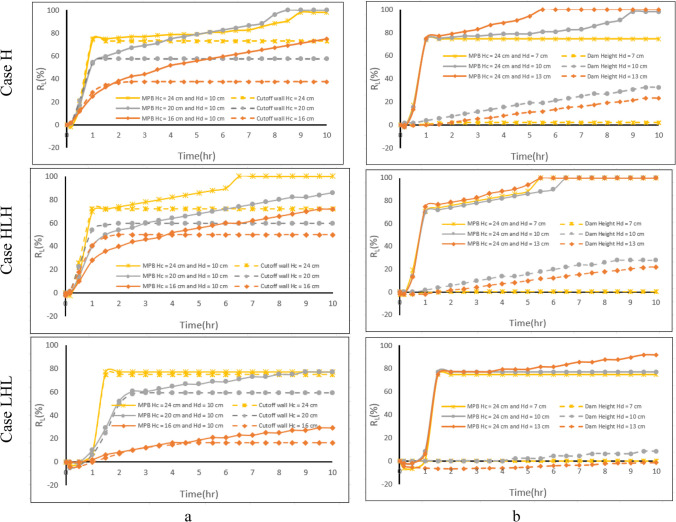
Comparison of MPB performance with a single physical barrier: cutoff wall (**a**) and subsurface dam (**b**)

**Fig.15:**
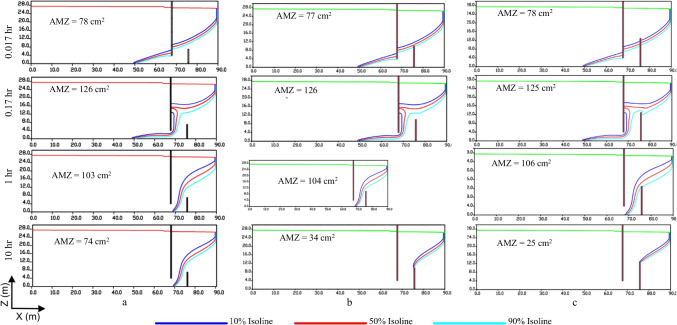
Dynamic changes of concentration field and the area of the mixing zone (AMZ) following the construction of the MPB with 7.0 cm (**a**), 10.0 cm (**b**), and 13.0 cm (**c**) subsurface dams’ height for the case H

**Fig. 16:**
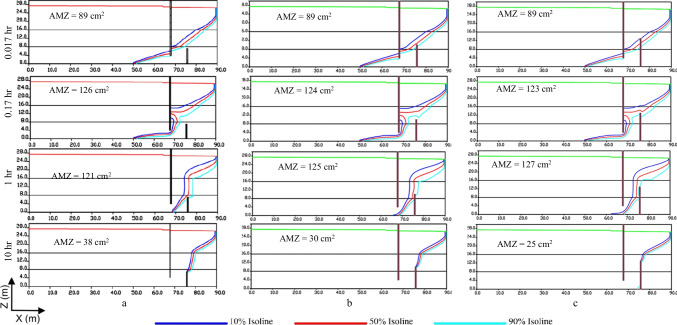
Dynamic changes of concentration field and the area of the mixing zone (AMZ) following the construction of the MPB with 7.0 cm (**a**), 10.0 cm (**b**), and 13.0 cm (**c**) subsurface dams’ height for the case HLH

**Fig. 17:**
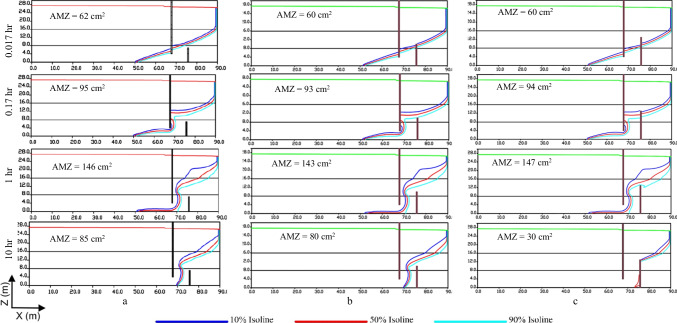
Dynamic changes of concentration field and the area of the mixing zone (AMZ) following the construction of the MPB with 7.0 cm (**a**), 10.0 cm (**b**), and 13.0 cm (**c**) subsurface dams’ height for the case LHL

**Fig. 18:**
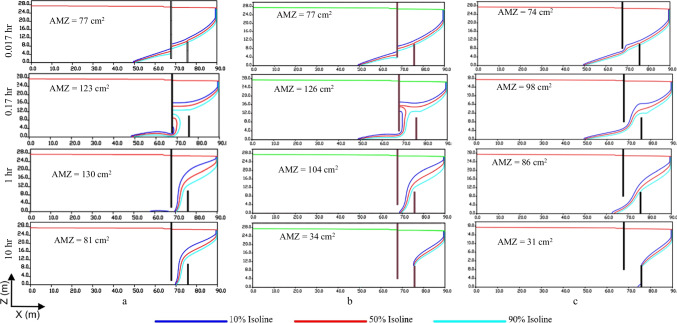
Dynamic changes of concentration field and the area of the mixing zone (AMZ) following the construction of the MPBs with 26 cm (**a**), 24 cm (**b**), and 20 cm (**c**) cutoff wall depths for the case H

**Fig. 19:**
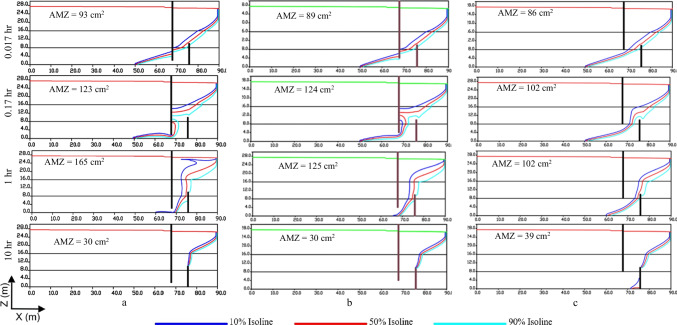
Dynamic changes of concentration field and the area of the mixing zone (AMZ) following the construction of the MPBs with 26 cm (**a**), 24 cm (**b**), and 20 cm (**c**) cutoff wall depths for the case HLH

**Fig. 20:**
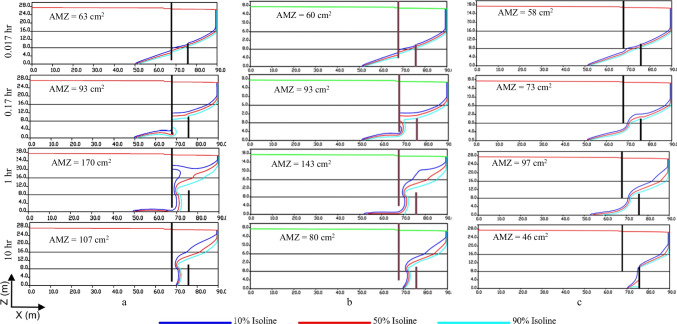
Dynamic changes of concentration field and the area of the mixing zone (AMZ) following the construction of the MPBs with 26 cm (**a**), 24 cm (**b**), and 20 cm (**c**) cutoff wall depths for the case LHL

**Fig. 21:**
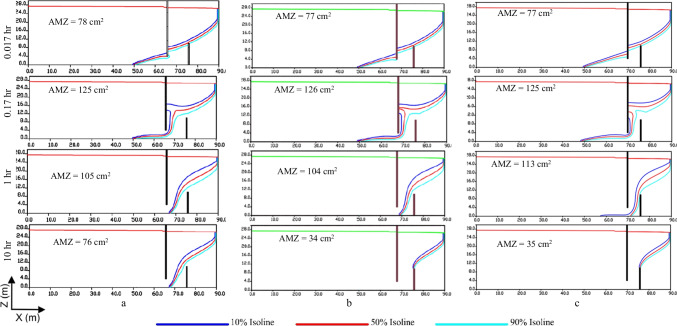
Dynamic changes of concentration field and the area of the mixing zone (AMZ) following the construction of the MPBs with 10 cm (**a**), 8 cm (**b**), and 6 cm (**c**) spacing for case H

**Fig. 22:**
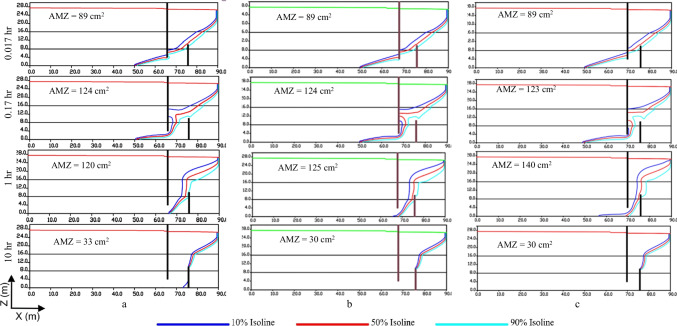
Dynamic changes of concentration field and the area of the mixing zone (AMZ) following the construction of the MPBs with 10 cm (**a**), 8 cm (**b**), and 6 cm (**c**) spacing for case HLH

**Fig. 23:**
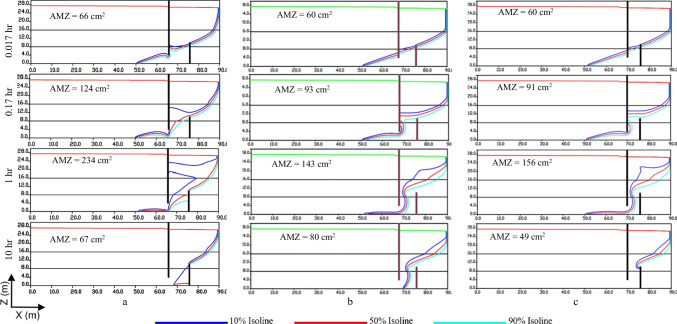
Dynamic changes of concentration field and the area of the mixing zone (AMZ) following the construction of the MPBs with 10 cm (**a**), 8 cm (**b**), and 6 cm (**c**) spacing for case LHL

The MPBs spacing and the depth of the cutoff wall remained consistent with the reference values, while various subsurface dame height values were modeled (Fig. 14a). The subsurface dam’s capability in removing residual saline water during the reference period was relatively low. The MPBs efficiency (R_L_) in removing residual saltwater were 2.06—3.30 and 2.07—3.55 times greater than that of the subsurface dam in cases H and HLH, respectively, and 8.25 times greater in case LHL. In another scenario, the cutoff wall was positioned in the same location as the MPB wall, while the reference values of subsurface dam height, MPB spacing, and various cutoff wall depths were examined in the simulations (Fig. 14b). The efficacy of MPBs (R_L_) in removing residual saline water was 38—100% and 39—44% greater than that of the cutoff wall in case H and case HLH, respectively, and 2.7–75% in case LHL. Significantly, the R_L_ result of the MPBs with H_c_^*^ = 0.394 in Fig. 11 was 52.9, 56.0, and 14.6%, whereas the comparable subsurface dam scenario (H_d_^*^ = 0.49 & H_d_ = 13 cm) in Fig. 14b was only 23.4, 22.0, and -1.5% for cases H, HLH, and LHL, respectively. This comparison highlights that, despite the shorter cutoff wall depth in the case of MPBs, they play a crucial role in expediting the removal of residual saline water. The use of a single physical barrier has limited potential to remove the residual saline water trapped upstream of the subsurface dam. The MPBs, on the other hand, demonstrate a higher removal efficacy and a sustained removal impact, which provides them with considerable benefits in the conservation and management of coastal GW resources.

## Conclusions

The impacts of mixed physical barriers (MPBs) on removing residual saline water in Multi-Layered unconfined coastal aquifers were investigated using a validated numerical model. In comparison to the experimental findings reported by Gao et al. (2021), the SEAWAT model demonstrated highly accurate predictions of the dynamic migration of the saltwater wedge during both intrusion and removal processes, yielding impressive R^2^ values of 0.99 and 0.97, respectively.

Three different aquifer configurations were investigated: a homogeneous reference aquifer (H) and two layered aquifers, namely HLH and LHL. The study aimed to assess the effectiveness of MPBs.The process of removing residual saline water can be divided into two distinct stages. Although the first stage represents only 10% of the total removal process duration, it exhibits substantial reductions in the length of the saltwater wedge (R_L_) with rates of 74.5%, 70.0%, and 68.8% for cases H, HLH, and LHL, respectively. In the second stage, the elimination of residual saltwater proceeds at a significantly slower pace across all cases until it reaches a steady state. These findings closely align with the research conducted by Gao et al. (2021), particularly in the context of a homogeneous aquifer scenario.

The effectiveness of the removal process following the construction of MPBs is strongly affected by the dimensions of the MPBs structure. To determine the most efficient MPBs dimensions, dimensionless parameters such as the subsurface dam height (H_d_^*^), cutoff wall depth (H_c_^*^), and MPB spacing (L_s_^*^) were employed. With an increase in the cutoff wall depth, the efficiency of removing residual saltwater also increased. When the cutoff wall depth was reduced from 24.0 to 12.0 cm, the repulsion ratio decreased by 47%, 44%, and 81% after 10 h for case H, case HLH, and case LHL, respectively. Notably, The MPB with H_c_* value of 0.85 (equivalent to H_c_ = 24 cm) exhibited the highest repulsion ratio in all the cases investigated.

Among the investigated cases, it appears that an MPB with H_d_^*^ equal to 0.5 (corresponding to H_d_ = 13 cm) offers the highest repulsion ratio. In case H, the removal time for residual saltwater was only 5.17 h, while for case LHL, it took 10 h to achieve an RL reduction of 91.7%. In the case of HLH, a dam height of 7 cm or higher (H_d_^*^ ≥ 0.264) proved optimal, resulting in a residual saltwater removal time of 5.5 h. Generally, the depth of the cutoff wall had a more pronounced impact on removal efficiency than the dam height. For all the investigated cases, MPBs with an L_s_^*^ value less than or equal to 0.15 demonstrated the maximum repulsion ratio. An MPB spacing of L_s_^*^ equal to 0.1 (corresponding to L_s_ = 4 cm) was found to be the optimal value, resulting in a rapid reduction in residual saltwater within 2.33 h for case H, 3.67 h for case HLH, and 4.33 h for case LHL.

In contrast to single physical barriers sharing the same physical properties, MPBs clearly demonstrated their advantages in the removal of residual saltwater. In cases H and HLH, the efficacy of MPBs (R_L_) in residual saline water removal was 2.06 to 3.30 and 2.07—3.55 times greater than that of the subsurface dam, respectively, while in case LHL, it was an impressive 8.34 times more effective. Moreover, in cases H and HLH, MPBs outperformed cutoff walls by 38% to 100% and 39% to 44%, respectively. In case LHL, MPBs exhibited a 2.7% to 75% advantage in residual saltwater removal. Even though the MPB's cutoff wall depth is short, it is critical to speed up the removal process of residual saltwater. These findings align closely with the research conducted by Gao et al. (2021), especially in the context of a homogeneous aquifer scenario.

The removal of residual saltwater by MPB in the current study was limited to a laboratory-scale aquifer, with the complete removal process unfolded within a few hours. This setup does not accurately reflect real-world conditions, where the natural removal of residual saltwater can extend over years to decades. Moreover, tidal fluctuations of seawater were not taken into account in this study. Real-world aquifers are characterized by complexity, potential anisotropy, and vulnerability to tidal and wave influences. Consequently, the removal of saltwater intrusion in natural aquifers is a much more intricate process, necessitating refinement of the current model to accurately represent these complexities and provide a more accurate representation of the real-world dynamics involved.

To better address the practicality of MPBs in real-world scenarios, future studies should consider a wider spectrum of aquifer characteristics, including depth, breadth, and lateral extent. Additionally, it is essential to undertake a thorough analysis of rainfall infiltration in upcoming research. Furthermore, the substantial impact of transient factors such as seasonal variations and sudden freshwater influxes on saltwater intrusion dynamics should be thoroughly investigated.

## Data Availability

The data that support the findings of this study are available on request from the corresponding author.

## Appendix A

In Appendix A, the dynamic evolution of concentration fields and the transformation of the Area of the Mixing Zone (AMZ) in response to the installation of the Mixed Physical Barriers (MPB) are examined. Diverse scenarios are the focus of this exploration, featuring variations in subsurface dam height (H_d_), cutoff wall depth (H_c_), and MPB spacing (L_s_). The aim here is to conduct an in-depth examination of how these critical parameters are influenced by the MPB construction, affecting the distribution of solute concentration within the aquifer and resulting in changes in the AMZ.

See Figs. 15, 16, 17, 18, 19, 20, 21, 22, and 23

## Abbreviation

AMZ: Area of the mixing zone
GW: Groundwater
Case H: Homogenous aquifer with high hydraulic conductivity
Case HLH: Stratified aquifer grouped from three layers with different hydraulic conductivities (high K – low K – high K)
Case LHL: Stratified aquifer grouped from three layers with different hydraulic conductivities (low K – high K – low K)
MPB: Mixed physical barriers
PB: Physical barrier
SWI: Seawater intrusion
